# Cost-effectiveness of GeneXpert and LED-FM for diagnosis of pulmonary tuberculosis: A systematic review

**DOI:** 10.1371/journal.pone.0205233

**Published:** 2018-10-29

**Authors:** Karuna D. Sagili, Malaisamy Muniyandi, Kayzad Soli Nilgiriwala, Kalpita S. Shringarpure, Srinath Satyanarayana, Richard Kirubakaran, Sarabjit S. Chadha, Prathap Tharyan

**Affiliations:** 1 International Union against Tuberculosis and Lung Disease, South East Asia Regional office, New Delhi, India; 2 National Institute for Research in Tuberculosis, ICMR, Chennai, India; 3 Tuberculosis Division, The Foundation for Medical Research, Mumbai, India; 4 Department of Preventive and Social Medicine, Medical College Baroda, Baroda, India; 5 Prof BV Moses Centre for Evidence- Informed Health Care, Christian Medical College, Vellore, India; Wadsworth Center, UNITED STATES

## Abstract

**Background:**

Early and accurate diagnosis of tuberculosis is a priority for TB programs globally to initiate treatment early and improve treatment outcomes. Currently, Ziehl–Neelsen (ZN) stain-based microscopy, GeneXpert and Light Emitting Diode-Fluorescence Microscopy (LED-FM) are used for diagnosing pulmonary drug sensitive tuberculosis. Published evidence synthesising the cost-effectiveness of these diagnostic tools is scarce.

**Methodology:**

PubMed, EMBASE and Cost-effectiveness analysis registry were searched for studies that reported on the cost-effectiveness of GeneXpert and LED-FM, compared to ZN microscopy for diagnosing pulmonary TB. Risk of bias was assessed independently by four authors using the Consensus Health Economic Criteria (CHEC) extended checklist. The data variables included the study settings, population, type of intervention, type of comparator, year of study, duration of study, type of study design, costs for the test and the comparator and effectiveness indicators. Incremental cost-effectiveness ratio (ICER) was used for assessing the relative cost-effectiveness in this review.

**Results:**

Of the 496 studies identified by the search, thirteen studies were included after removing duplicates and studies that did not fulfil inclusion criteria. Four studies compared LED-FM with ZN and nine studies compared GeneXpert with ZN. Three studies used patient cohorts and eight were modelling studies with hypothetical cohorts used to evaluate cost-effectiveness. All these studies were conducted from a health system perspective, with four studies utilising cost utility analysis. There were considerable variations in costing parameters and effectiveness indicators that precluded meta-analysis. The key findings from the included studies suggest that LED-FM and GeneXpert may be cost effective for pulmonary TB diagnosis from a health system perspective.

**Conclusion:**

Our review identifies a consistent trend of the cost effectiveness of LED-FM and GeneXpert for pulmonary TB diagnosis in different countries with diverse context of socio-economic condition, HIV burden and geographical distribution. However, all the studies used different parameters to estimate the impact of these tools and this underscores the need for improving the methodological issues related to the conduct and reporting of cost-effectiveness studies.

## Introduction

Tuberculosis (TB) remains a leading cause of death worldwide. Globally, 10.4 million new cases were reported by WHO in 2016 [1]. India is amongst the six countries that accounted for 60% of the new cases. The Sustainable Development Goals (SDGs) and the End TB Strategy aim to end the global TB epidemic and reduce TB deaths by 90% and TB incidence by 80% in 2030 [2]. Though TB treatment averted 49 million deaths globally between 2000 and 2015, diagnostic gaps persist [1]. The WHO 2015 report estimates that about 37% of the cases were undiagnosed or not reported [3]. The potential transmission through people with undiagnosed TB to their contacts poses a serious public health problem. Hence, early and accurate diagnosis of TB is now the top priority of national TB programs globally. Delayed diagnosis contributes to continued transmission, poor health outcomes and distress to the patient and the family [4]. Early diagnosis is expected to lead to early treatment initiation and hence better outcomes. Improved diagnostic tools may facilitate early diagnosis and reduce the direct costs of the diagnostic burden on patients and family [5,6]. Currently, Ziehl–Neelsen (ZN) stain-based microscopy, GeneXpert and Light Emitting Diode-Fluorescence Microscopy (LED-FM) are widely used diagnostic tools for drug-sensitive pulmonary tuberculosis by National TB programmes in high burden countries.

### Current diagnostic tools

Sputum microscopy has been the main tool for TB diagnosis for nearly a century; followed by sputum culture, which is considered as the gold standard. However, these two tools have their inherent limitations viz. low sensitivity for microscopy and prolonged duration to obtain culture test results. ZN stain-based smear microscopy, using Carbol-fuchsin, Ziehl-Neelsen or Kinyoun acid-fast stains with an artificial light source or reflected sunlight, is widely used to detect acid fast bacillus (AFB). However, it has variable sensitivity (78%; 95% CI 32% to 89%) though it has higher specificity (98%; 95% CI 85% to 100%) for the diagnosis of pulmonary sputum smear-positive TB [7]. Sputum smear microscopy has been relied upon as a primary diagnostic tool in resource limited settings as it is cheaper with minimal required biosafety standards [3]. Thus, it continues to be the routine diagnostic method for pulmonary TB in countries like India [8]. It is simple and inexpensive, and at the same time allows rapid detection of the most infectious cases of pulmonary TB. It can be used for TB diagnosis at the peripheral level as well [9]. Though highly specific [8], it is limited by its low sensitivity (further reduced in patients with extra-pulmonary TB, children and HIV/TB co-infected patients).

GeneXpert (Cepheid, Sunnyvale, USA) is a newer molecular test that detects DNA of TB bacteria in sputum samples (pooled sensitivity– 98%; 95% CI 85%-92% and specificity 99%; 95% CI 98%-99%) and also detects resistance to Rifampicin within two hours. This simplifies molecular testing with fully integrated and automated sample preparation, compared to the procedure and time required for amplification and detection by real-time PCR [7, 10]. The cost of GeneXpert per cartridge is US$17 universally except for some high TB burden and low income countries which receive a discounted cost of about US$10 [11]. It was reported that implementation of GeneXpert would result in a three-fold increase in the diagnosis of patients with drug-resistant TB and a two-fold increase in the number of HIV-associated TB cases [12]. It is also useful for diagnosing smear negative specimens considering the lack of accuracy of smear microscopy. While testing single sputum samples in a prospective study of people suspected to have TB, GeneXpert detected 98% to 100% of those with sputum smear-positive disease and 57% to 83% of those with smear negative disease [7]. Countries like South Africa are offering this test upfront for TB diagnosis, and India is also scaling up its GeneXpert services across the country.

Around the same time as the introduction of GeneXpert, evidence on the efficacy of the LED-FM was provided by the WHO in 2009. Sensitivity of LED-FM is comparable to that of conventional fluorescence microscopy and it surpasses that of conventional Ziehl–Neelsen microscopy by an average 10%. Conventional fluorescence microscopy replacement with LED-FM has been recommended by WHO [8, 9]. A retrospective cohort study on cost utility of LED-FM showed it to be a cost effective intervention in diagnosis of pulmonary TB in India with an Incremental Cost-effectiveness Ratio (ICER) of US$14.64 per disability-adjusted life-year (DALY) averted [13].

Expenditure for TB program in India was 6398.6 million rupees (US$ 98.47 million) in 2015–16 [14]. Low and middle-income countries fell short of almost US$ 2 billion of the US$ 8.3 billion needed in 2016, which was required to combat the TB epidemic [1]. This amount excludes the funding required for research and development. Thus, *“Global actions and investments fall far short of those needed to end the global TB epidemic”* [14].

There are several direct and indirect costs entailed to delayed diagnosis and treatment of TB, which can be averted with early and prompt diagnosis [14, 15]. Costs are usually described in monetary units, while effects can be measured in terms of health status or another outcome of interest. The incremental cost-effectiveness ratio (ICER) summarizes the additional cost per unit of health benefit gained in switching from one medical intervention to another [16]. A common application of the ICER is in cost-utility analysis, in which case the ICER is synonymous with the cost per quality-adjusted life year (QALY) gained, where

ICER=(Costofnewdiagnostic–Costofstandardcare)/(Effectivenessofnewdiagnostic–Effectivenessofstandardcare).

Considering the challenges in TB diagnosis and the limited resource, there is a need of a cost-effective tool as a priority that is highly sensitive and specific to be used in resource poor settings. Though there are recent systematic reviews on diagnostic accuracy of newer tools such as GeneXpert, these reviews do not report incremental costs and hence have limitation in guiding decision makers. A test having a good value doesn’t always mean it is affordable or feasible [15, 17]. It is important for the national TB programs to know what additional health unit benefits would accrue, if any, by changing a diagnostic tool and what additional costs this would incur. In the absence of any systematic reviews reporting on the incremental cost-effectiveness of the newer diagnostic tools, we undertook a systematic review to evaluate the incremental cost-effectiveness of GeneXpert and LED-FM in comparison with ZN microscopy for the diagnosis of smear-positive pulmonary TB.

## Methods

This systematic review was conducted following the PRISMA guidelines [18] (S1 Table). The review protocol is registered at the Prospero registry (Registration No. CRD42016043333) [19]. The objective was to compare the incremental cost-effectiveness of GeneXpert and LED-FM with ZN smear microscopy in the diagnosis of smear-positive pulmonary TB. Though we had initially planned to include Chest X-ray as one of the diagnostic tests evaluated, we excluded it for this review due to the lack of studies providing data comparing cost-effectiveness of Chest X-ray with ZN smear microscopy. Below is the PICO question for this review:

P—(Participants/population): Presumptive pulmonary TB patients undergoing diagnostic evaluation

I–(Interventions): GeneXpert, LED FM microscopy

C–(Comparator): ZN microscopy

O–(Outcome measures): To find out the incremental cost-effectiveness ratio (ICER) for GeneXpert and LED FM in comparison to ZN sputum microscopy from a health system perspective.

### Selection criteria

#### Types of studies

All types of studies (cross-sectional, observational, cohort, modelling, economic evaluation) that reported on cost-effectiveness of ZN microscopy, GeneXpert and LED-FM for pulmonary TB diagnosis were included.

#### Study population

Any person presumed to have pulmonary TB who was undergoing diagnostic evaluation irrespective of co-morbidities like infection with the Human Immunodeficiency Virus (HIV).

#### Diagnostic tests

Studies comparing GeneXpert with ZN microscopy and LED-FM in comparison to ZN microscopy for the diagnosis of pulmonary TB, with data provided for costs as well as for effectiveness. Studies reporting cost-effectiveness of GeneXpert or LED-FM but using a comparator other than ZN microscopy were excluded. Studies reporting only costs and not reporting an effectiveness indicator were also excluded.

#### Outcome measures

The primary outcome measure was incremental cost-effectiveness ratio (ICER) for GeneXpert and LED-FM compared to ZN microscopy. The secondary outcomes were additional case detection, cure rate, and time to initiate treatment post-diagnosis. The ICER [20] is an informative measure generated from economic/cost analysis and represents the ratio of the difference in cost between two health interventions to the difference in outcomes between the two interventions. Since the ICER summarizes the additional cost per unit of additional health benefit gained in switching from one health intervention to another, it serves as an important measure to guide decisions about allocating scarce resources across competing medical interventions.

### Search strategies

We searched PubMed, EMBASE and Cost-effectiveness analysis registry [21] using the search strategies detailed in S2 Table. We also searched the Cochrane database [22]. The searches were conducted in April 2017, and finalised on 24^th^ April 2017. The search has been updated till July 2018.

### Selection of studies

The abstracts for all papers retrieved by the search that were considered relevant to this review were uploaded in the Rayyan software [23] and screened for duplicates. After removing duplicates, the remaining abstracts were screened independently for relevance by four authors (KDS, MM, KSN, and KSS). Conflicts were resolved through discussions among the four investigators. Full texts of articles identified as relevant were obtained. When full texts of studies mentioned the cost-effectiveness as a key objective, but did not report an effectiveness indicator, they were excluded.

### Data extraction

Data from the included studies were extracted into a data extraction form independently by MM and KSN. The data variables included the study settings, population, type of intervention, type of comparator, year of study, duration of study, type of study design, costs for the test and the comparator, effectiveness indicators and others. A sample extraction form is given in the supplementary material (S1). Wherever the key data was missing, we contacted the authors; however, there was no response from the authors. In case of disagreements, it was discussed with KDS and KSS and extraction was completed after obtaining consensus.

#### Risk of bias assessment

MM and KSN assessed the risk of bias for each included study using the Consensus Health Economic Criteria (CHEC) extended checklist [24]. The checklist consists of 20 items with positive responses scored 1 and negative responses scored 0. The total score for each item was summed and converted to a percentage with the range of scores ranging from zero to 100. The total CHEC score for each study was categorized into four grades: low, moderate, good and excellent using cut-off value of ≤50, 51–75, 76–95 and >95, respectively. Higher scores denote lower risk of bias.

## Results

### Study selection

**Fig 1:**
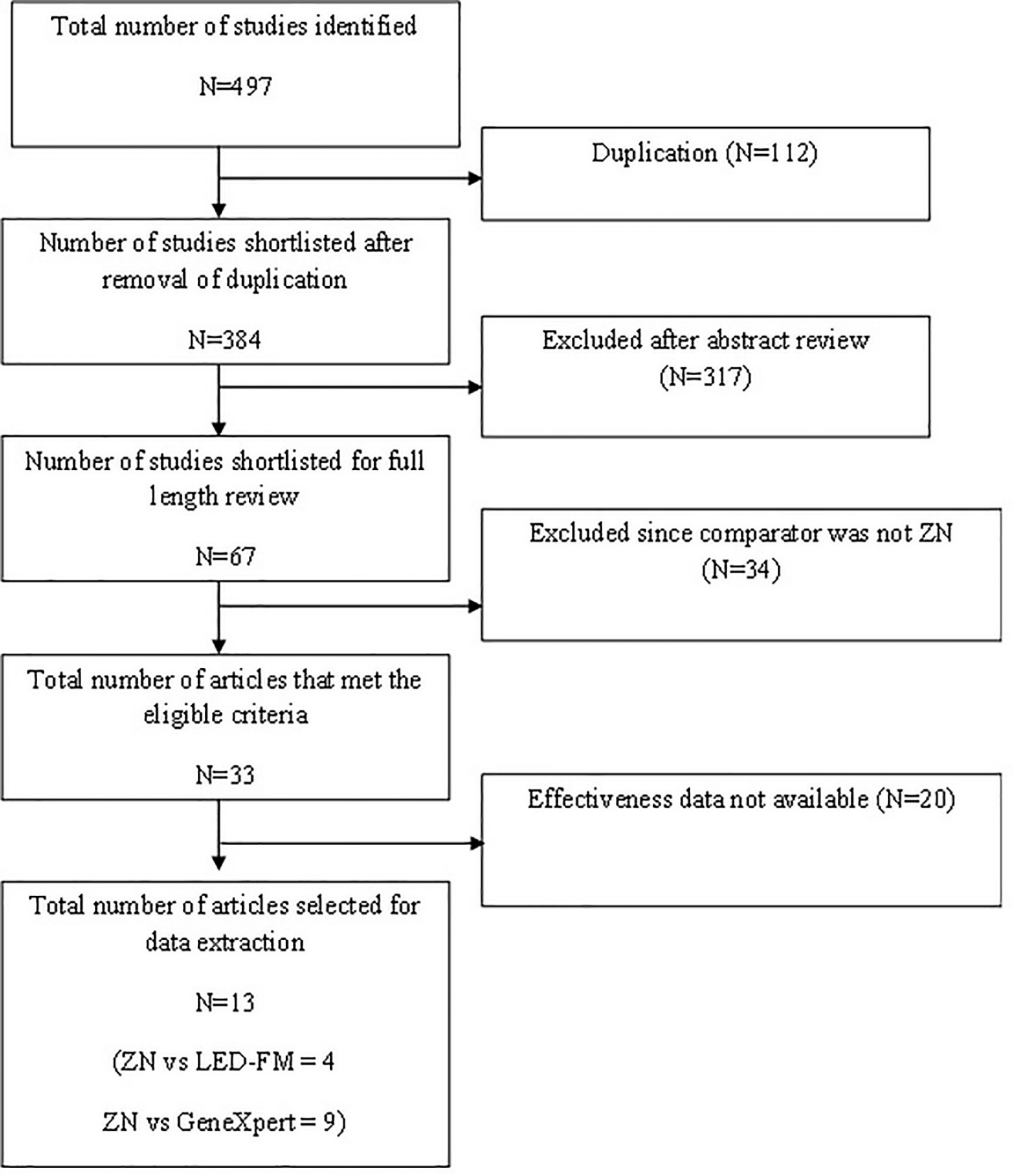
Flow diagram indicating the process of selecting the studies for this systematic review on cost-effectiveness of tolls to diagnose pulmonary TB.

Fig 1 depicts the study selection process. The search yielded 497 studies that had reportedly assessed the cost-effectiveness of GeneXpert and LED-FM. Of these 384 studies were shortlisted after excluding 112 duplicates. After the review of abstracts, 67 studies were retained for evaluation of full papers. Thirty-four studies were further excluded since they did not have a ZN smear microscopy comparator [S3 Table]. Of the remaining 33, twenty studies were excluded due to lack of effectiveness data [S4 Table]. Finally this review included 13 studies from which data were extracted; four of the included studies compared LED-FM with ZN [13, 25, 26, 27] and seven studies compared ZN with GeneXpert [28, 29, 30, 31, 32, 33, 34, 35, 36].

### Characteristics of included studies

Out of the 13 studies, seven were conducted in Africa [25, 28, 31, 32, 34, 35, 36] of which four were from South Africa [25, 31, 32, 36], one was a multi-centric study which included Botswana, Lesotho, Namibia, South Africa and Swaziland [34], one from Zambia [28] and one from Ethiopia [35] (Table 1). Four studies were conducted in Asia with one each from India, China, Hong Kong and Thailand [13, 26, 27, 30]. Two studies were from the Americas, one each from USA and Brazil [33, 29]. All the studies except for the one from USA were conducted in low and middle-income countries. Ten studies were conducted within the time period of 2011 to 2017 [13, 25, 27, 28, 29, 30, 31, 32, 33, 34, 35, 36]. Seven studies were conducted in an urban or peri-urban setting [13, 25, 30, 31, 32, 33, 35], while others did not mention the study setting clearly (Table 1). Three studies used the real patient cohorts [25, 26, 27] and eight used modelling studies with hypothetical cohorts to evaluate the cost-effectiveness of different diagnostic tools for pulmonary TB diagnostics.

**Table 1:** Characteristics of the 13 studies included in the review.

Sr. No.	First Author, Year	Country	Setting	Funding Source	Type of Economic Evaluation	Target Population	Comorbidities	Study Perspective	Reporting of ICER	Study Design	Time Horizon (years)	Sensitivity analysis
**LED-FM vs ZN microscopy**												
1	Whitelaw, 2011	South Africa	Urban	European Commission & Canadian Institute of Health Research	CEA	Adults	HIV	Health system	NR	Cross-sectional	1	NR
2	Kelly, 2015	India	Urban	TB Reach Initiative	CUA	Adults	NR	Health system	Yes	Cohort	1	One-way PA
3	Sohn, 2009	Thailand	NR	USAID & CDC	CEA	NR	NR	Health system	NR	Cross-sectional	0.25	NR
4	Xia, 2013	China	NR	BMGF	CEA	NR	NR	Health system	No	Cross-sectional	1	NR
**GeneXpert vs ZN microscopy**												
5	Mishra, 2012	Zambia	NR	NR	CEA	NR	HIV	Health system	Yes	Cohort	NR	NR
6	Pinto, 2016	Brazil	NR	Bill & Melinda Gates Foundation	CEA	NR	HIV	Health system	Yes	Cohort	NR	Monte Carlo simulation
7	You, 2015	Hongkong	Urban	No funding	CUA	Adults	NR	Health system	Yes	Cohort	10	Monte Carlo simulation
8	Jha, 2016	South Africa	Urban	Frank & Kathleen Polk Assistant Professorship in Epidemiology	CEA	Adults	NR	Health system	Yes	Model	NR	One-way PA
9	Andrews, 2012	South Africa	Peri-Urban	NatioNDl Institute of General Medical Sciences	CUA	NR	HIV	Health system	Yes	Cohort	NR	Two-way PA
10	Millman, 2013	USA	Urban	American Lung Association, UCSF-GIVI Centre for AIDS Research, National Institutes of Health & NDtioNDl Centre for Research Resources	CBA	NR	NR	Health system	No	Cohort	1	Monte Carlo simulation
11	Menzies, 2012	Botswana, Lesotho, Namibia, South Africa & Swaziland	NR	UNITAID & MGH Program in Cancer Outcome & Training	CUA	NR	HIV	Health system	Yes	Model	10	Monte Carlo simulation
12	Vassall, 2017	South Africa	NR	**Bill & Melinda Gates Foundation**	CUA	Adults	HIV	Health system	Yes	cluster-randomised trial	1	One-way PA
13	Tesfaye A, 2017	Ethiopia	Urban	USAID/TB CARE	CUA	NR	HIV	Health system	Yes	observational quantitative modeling	10	One-way PA

NR–Not reported; PA–Probabilistic analysis

All these studies were conducted from a health system perspective with seven studies utilising cost utility analysis. Four used Disability Adjusted Life Years (DALY) [13, 34, 35, 36], one [30] used Quality Adjusted Life Years (QALY) and one [31] used years of life saved (YLS) as indicators, all these being standard indicators for cost-effectiveness analysis. There were also studies that used other indicators like time duration per slide for diagnosis [25, 26, 27], additional cases diagnosed [29, 31], TB cases averted [28] and reduction in duration of hospitalisation as an effectiveness indictor [33]. Five studies mentioned the target population as adults and seven studies also included patients with HIV co-infection [13, 25, 30, 31, 35, 36]. Out of 13 studies ICER value was reported by nine studies [13, 28, 29, 30, 31, 32, 33, 34, 35, 36]. Nine studies mentioned their time horizon ranging from 3 months to ten years [13, 25, 26, 27, 30, 33, 34, 35, 36]. Eleven studies were funded by international agencies like Stop TB, USAID and DFID [13, 25, 26, 27, 29, 31, 32, 33, 34, 35, 36] and the remaining two did not mention about funding [28, 30].

### Quality of included studies

Table 2 summarises the appraisal of reporting quality for each study using the Extended CHEC checklist. Of the 13 studies, seven studies were of moderate quality while five were of good quality, indicating lower risk of bias. One study was graded as low score however it was decided to include this study owing to less number of studies qualifying for review purpose. Overall, four studies fulfilled ≥80% of the 20 items as per the checklist [29, 31, 33, 34]. Two studies [31, 32] did not mention the time horizon over which costs and consequences were being evaluated.

**Table 2:** Consensus Health Economic Criteria (CHEC) extended checklist for quality assessment of the included studies.

Sr. No.	Checklist question	Whitelaw 2011	Kelly* 2015	Sohn 2009	Xia 2013	Mishra 2012	Pinto 2016	You 2015	Jha 2016	Andrews 2012	Millman 2013	Menzies 2012	Vassall 2017	Tesfaye 2017	Total (% of Yes)
1	Is the study population *clearly* described?	1	0	0	0	0	0	1	1	0	1	0	1	0	38
2	Are competing alternatives *clearly described*?	1	1	1	1	1	1	1	1	1	1	1	1	1	100
3	Is a *well-defined* research question posed in answerable form?	1	1	1	1	1	1	1	1	1	1	1	1	1	100
4	Is the economic study design appropriate to the stated objective?	1	1	1	1	1	1	1	1	1	1	1	1	1	100
5	Are the structural assumptions and the validation methods of the model properly reported?	0	1	0	0	0	1	1	0	1	1	1	1	1	62
6	Is the chosen time horizon *appropriate* in order to include relevant costs and consequences?	1	1	1	1	0	1	1	0	0	1	1	1	1	77
7	Is the actual perspective chosen appropriate?	1	1	1	0	0	1	1	1	1	1	1	1	1	85
8	Are *all important and relevant* costs for each alternative identified?	1	0	1	1	0	1	1	1	1	1	1	1	1	85
9	Are all costs measured *appropriately* in physical units?	1	0	1	1	0	1	0	1	0	1	1	1	1	69
10	Are costs valued *appropriately*?	0	0	1	1	0	1	0	1	0	1	1	1	1	62
11	Are *all important and relevant* outcomes for each alternative identified?	0	0	1	1	1	1	1	1	1	1	1	1	1	85
12	Are all outcomes measured appropriately?	0	0	0	1	1	1	1	1	1	1	1	1	0	69
13	Are outcomes valued *appropriately*?	1	1	1	1	0	1	0	0	0	0	0	1	0	46
14	Is *an appropriate* incremental analysis of costs and outcomes of alternatives performed?	0	1	0	0	1	1	1	1	1	0	1	1	0	62
15	Are all future costs and outcomes discounted *appropriately*?	0	0	0	0	0	1	1	1	1	0	1	1	1	54
16	Are all important variables, whose values are uncertain, *appropriately* subjected to sensitivity analysis?	0	1	0	0	0	1	1	1	1	1	1	1	1	69
17	Do the conclusions follow from the data reported?	1	1	1	1	1	1	1	1	1	1	1	1	1	100
18	Does the study discuss the generalizability of the results to other settings and patient/client groups?	0	1	1	0	0	1	0	1	1	1	1	1	1	69
19	Does the article/report indicate that there is no potential conflict of interest of study researcher(s) and funder(s)?	1	1	0	0	0	1	1	0*	0	0*	0*	1	1	46
20	Are ethical and distributional issues discussed *appropriately*?	0	1	1	1	0	1	0	1	0	1	0	0	0	46
	**% of Yes**	**55**	**65**	**65**	**60**	**35**	**95**	**75**	**80**	**65**	**80**	**80**	95	75	
	**Overall Quality**	**Moderate**	**Moderate**	**Moderate**	**Moderate**	**	**Good**	**Moderate**	**Good**	**Moderate**	**Good**	**Good**	Good	Moderate	

* Conflict of interest present

** Not categorised due to lack of information

Two studies did not clearly state the funding sources and conflict of interest [28, 30]. Out of 13 studies five studies did not include all costs components and these were not valued appropriately [13, 25, 28, 30, 32].

### Incremental Cost-Effectiveness of LED FM compared with ZN microscopy

The sample size in the four studies [13, 25, 26, 27] comparing LED FM and ZN microscopy ranged from 345 to 21450 for test and from 345 to 14,300 for comparator. One of the studies used decision tree modelling analysis [13], while the cost indicator for all the four studies was average cost per smear. The cost for LED-FM ranged from USD 0.31 to 1.97 and the cost for ZN ranged from USD 0.21 to 2.2. The effectiveness indicator used in three of the studies [25, 26, 27] was time per reading of one slide in minutes, which ranged from 1–2 minutes for LED-FM and 2.4–3.4 minutes for ZN microscopy. The ICER values for these studies were calculated in this review (Table 3). The effectiveness indicator used in one of the study [13] was DALYs, which was 27.45 for LED-FM and 40.84 for ZN microscopy and the ICER value was 14.64 (Table 3). The range of cost-effectiveness ratio observed maybe due to different study settings, populations and methodology used.

**Table 3:** Description of cost-effectiveness analyses reported in the included studies.

Sr. No.	First Author, Year	Country	Economic Evaluation Type	Sample size (Test)	Sample size (ZN)	Model Type	Year Cost	Cost Indicator	Cost of Test	Cost of ZN	Effectiveness Indicator	Effectiveness-Test	Effectiveness-ZN	ICER	ICER Threshold	Sensitivity Analysis	Conclusion
**LED-FM vs ZN microscopy**																	
1	Whitelaw, 2011	South Africa	CEA	345	345	NA	2009–10	Average cost per smear	1.63	2.1	Time per slide (min)	1.8	2.5	0.67*	NR	NR	LED-FM microscopy is cheaper
2	Kelly, 2015	India	CUA	21,450	14,300	Decision Tree	2011–12	Average cost per smear	0.31	0.21	DALYs	27.45	40.84	14.64	1489	One-way PA	LED-FM is cost effective at high load settings
3	Sohn, 2009	Thailand	CEA	30/day	30/day	NA	2007	Average cost per smear	1.03	1.16	Time per slide (min)	1	2.4	0.09*	NR	NR	LED-FM is cost-effective in resource limited settings
4	Xia, 2013	China	CEA	11,276	11,276	NA	2013	Average cost per smear	1.97	2.2	Time per slide (min)	2	3.4	0.16*	NR	NR	LED-FM is cost-effective in peripheral laboratories
**GeneXpert vs ZN microscopy**																	
5	Mishra, 2012	Zambia	CEA	NR	NR	Decision Tree	NM	Cost per case detected	108.9	75.74	TB cases averted	NR	NR	252	NR	NR	-
6	Pinto, 2016	Brazil	CUA	NR	NR	Decision Tree	2014	Average cost per sample	14.69	3.08	Additional case diagnosed (%)	3.9	NR	643	11,000	Monte Carlo simulation	Single-sample GeneXpert testing can replace 2-sample sputum smear microscopy test
7	You, 2015	Hongkong	CUA	NR	NR	Decision Tree	2014	Average cost per sample	128	7.5	QALYs	NR	NR	99	50,000	Monte Carlo simulation	Single sample GeneXpert testing during initial assessment of hospitalized patients is highly cost-effective
8	Jha, 2016	South Africa	CEA	1,009	NR	Economic Model	2015	Average cost per sample	14.45	1.59	Additional case diagnosed	NR	NR	1,927	2,000	One-way PA	GeneXpert is likely to be highly cost-effective where the level of empiric TB diagnosis is low
9	Andrews, 2012	South Africa	CUA	NR	NR	CEPAC	2010	Average cost per sample	21.6	4.6	Years of life saved (YLS)	NR	NR	5,100	21,300	Two-way PA	Two-sample GeneXpert testing is very cost-effective for screening all individuals initiating ART
10	Millman, 2013	USA	CBA	1,358	1,381	Decision Tree	2011	Average cost per sample	218	15	Reduction in hospitalization	NR	NR	101.5*	NR	Monte Carlo simulation	GeneXpert provides substantial savings to hospitals in high income countries by reducing overall length of stay
11	Menzies, 2012	Botswana, Lesotho, Namibia, South Africa & Swaziland	CUA	8,92,000	8,92,000	Dynamic compartmental model	2011	Average cost per sample	45	31	DALYs	NR	NR	959	1,000	Monte Carlo simulation	GeneXpert has the potential to produce a substantial reduction in TB morbidity and mortality
12	Vassall, 2017	South Africa	CUA	2324	2332	NA	2012	Average cost per participant	168.79	160.64	DALYs	NR	NR	16.37	NR	One-way PA	Xpert introduction in South Africa was cost-neutral
13	Tesfaye A, 2017	Ethiopia	CUA	54000	113000	discrete-event simulation	2014	annualized cost per DALY averted	NR	NR	DALYs	NR	NR	127	690	One-way PA	Xpert is considered cost effective

* ICER calculated; NA = Not Applicable; NR = Not Reported

### Incremental cost-effectiveness of GeneXpert compared to ZN microscopy

Sample size in the seven studies comparing GeneXpert and ZN microscopy ranged from 1009 to 8,92,000 for test and comparator. Four studies [13, 28, 29, 30] used decision tree modelling analysis, one study used Cost Effectiveness of Preventing AIDS complications (CEPAC) model [32] and one study used dynamic compartmental modelling [34]. Six of the studies used average costs per sample as the cost indictor [24, 25, 26, 27, 28, 29] and one study used cost per case detected [28]. The average cost per sample for GeneXpert ranged from USD 14.45 to 218 and the cost for ZN ranged from USD 1.59 to 31. In one study, the average cost per case detected was USD 108.9 for GeneXpert and the cost for ZN was USD 75.74 [28]. These studies used different effectiveness indicators such as TB cases averted, additional case diagnosed, QALYs, DALYs, YLs and reduction in hospitalisation and ICER values were calculated accordingly (Table 2). Except in one study [28], sensitivity analysis was done using either Monte Carlo Simulation (4 studies [29, 30, 33, 34]), one way (one study, [31]) or two-way probabilistic analysis (one study) [32].

### Different components of costs used for costs calculation

For cost calculation, broadly six components such as laboratory space, staff, training, equipment, consumables and overheads were used in the studies (Table 4). Out of 13 studies none included all the six components. Additionally, one study included waste disposal [27] and one study included transportation cost components [34]. There was variation in inclusion of different costs components. Though the reasons for this variation are not clear, individual studies perceived the importance of each component differently, and it may depend on their outcome of interest or the effectiveness indicator.

**Table 4:** Key Cost components reported by the studies included in the review.

Sr. No.	First Author, Year	Country	Lab space	Staff	Training	Equipment	Consumables	Overheads	Disposal	Transport	Checkmarks	Cost of Test	Cost of ZN
1	Whitelaw, 2011	South Africa	✓	✓	✕	✓	✓	✓	✕	✕	**5**	1.63	2.1
2	Kelly, 2015	India	✕	✕	✓	✓	✓	✕	✕	✕	**3**	0.31	0.21
3	Sohn, 2009	Thailand	✓	✓	✕	✓	✓	✓	✕	✕	**5**	1.03	1.16
4	Xia, 2013	China	✓	✓	✕	✓	✓	✓	✓	✕	**6**	1.97	2.2
5	Mishra, 2012	Zambia	-	-	-	-	-	-	-	-	**0**	108.9	75.74
6	Pinto, 2016	Brazil	✕	✓	✕	✓	✓	✓	✕	✕	**4**	14.69	3.08
7	You, 2015	Hongkong	✕	✓	✕	✕	✓	✕	✕	✕	**2**	128	7.5
8	Jha, 2016	South Africa	✓	✓	✕	✓	✓	✓	✕	✕	**5**	14.45	1.59
9	Andrews, 2012	South Africa	✕	✓	✕	✕	✓	✕	✕	✕	**2**	21.6	4.6
10	Millman, 2013	USA	✕	✓	✕	✓	✓	✓	✕	✕	**4**	218	15
11	Menzies, 2012	Botswana, Lesotho, Namibia, South Africa & Swazil&	✕	✓	✕	✓	✓	✓	✕	✓	**5**	45	31
12	Vassall, 2017	South Africa	✕	✓	✓	✓	✓	✕	✕	✓	**5**	168.79	160.64
13	Tesfaye, 2017	Ethiopia	✕	✓	✓	✓	✓	✓	✕	✕	**5**	NR	NR

Additional health system costs per year over 10 years is used for different algorithms, to calculate ICER value, hence cost per test is not reported. NR = Not reported

## Discussion

To the best of our knowledge, this is the first systematic review to synthesize the evidence of cost-effectiveness of LED-FM and GeneXpert in comparison to ZN microscopy for pulmonary TB diagnosis. The review also appraised the reporting quality of the published evidence. The key findings from the included studies suggest that the new diagnostic tools LED-FM and GeneXpert are very cost effective for pulmonary TB diagnosis from a health system perspective, even though they are not cost saving to the health system. The evidence from 11 countries, with majority of them having high TB burden shows that these new tools are cost effective irrespective of their economic condition, HIV burden and geographical distribution.

For LED-FM, only one out of four studies reported ICER values and, for the remaining three studies, ICER was calculated using the data provided [13, 27–31, 33]. Three studies used average time per slide reading as the effectiveness indicator, while one study used DALYs. The average time taken to read one ZN stained slide is 2.8 (±0.4) minutes. By using the new tool LED-FM this can be reduced to 1.6 (±0.4) minutes, with an additional cost of less than one USD. This additional costs fall within the ‘willingness to pay threshold’ of each country. Hence, this tool is cost-effective to diagnose pulmonary TB. One study from India reported the long-term impact in terms of DALYs which indicated additional cost of USD 14.64 to avert one DALY. This additional cost is less than the national ‘willingness to pay threshold’ of USD 1489 for India [13]. Apart from being cost-effective, LED-FM is user-friendly and more acceptable among technicians. It can also be extended to other infectious disease diagnosis like malaria and trypanosomiasis, reducing the costs involved in providing integrated laboratory services [34]. Considering this factor, LED-FM could possibly be more cost-effective in countries with high double burden of TB and malaria.

GeneXpert studies included in this review used different short term (additional case diagnosed, reduction in duration of hospitalisation) and long term (TB case averted, QALYs, DALYs and YLS) effectiveness indicators. There was a huge variation in terms of cost per unit of health benefit which could be due to the different effectiveness indicators, year of study and the subsidised rate of GeneXpert cartridges to high burden countries. For instance, it was observed that health system will have to pay at least USD 1927 for a short-term benefit of additional TB case diagnosed if GeneXpert is preferred in South Africa [30]. This additional cost is very close to the maximum of willingness to pay threshold USD 2000. However, another study from South Africa in 2012 [31] reported an ICER of USD 5100 to save one life-year which is a long-term benefit. This also is within the willingness-to-pay threshold of USD 21,300.

This review observed that the included studies analysed effectiveness in terms of different indicators. Results of these studies conclude that implementation of GeneXpert will increase case detection, reduce duration of hospitalisation, gain QALYs, reduce DALYs and save additional years of lives. Also, the investment is within the willingness to pay threshold to avert TB cases. However, most of the studies have not included the sensitivity and specificity of the test in the calculation. Additional to these benefits, GeneXpert can diagnose rifampicin resistance, contributing to early diagnosis of TB as well as rifampicin resistance TB, early treatment initiation and indirectly reduce transmission in the community. However, none of these factors have been considered in cost calculation in the included studies. Thus, the costs calculated may have been underestimated. It is possible that if these studies include the above mentioned factors, GeneXpert may prove to be even more cost-effective.

Furthermore, the current review assessed the reporting quality of the studies using the CHEC checklist which consists of 20 items. It was observed that none of the studies included all cost components which resulted in under estimation of total costs. This indicates variability in the methods used to determine the costs involved in the diagnosis of pulmonary TB. Additionally, none of the studies are based on randomised controlled trials which provide rigorous comparison. Majority of the studies included limited cost components such as consumables and staff costs to calculate costs. Similarly, the effectiveness indicators varied in different studies due to which meta-analysis was not possible in this current review. Sensitivity analysis was performed in almost all the GeneXpert studies. None of the studies mentioned about the methods of calculations of QALYs, DALYs and YLS. This review provides the way forward to compare the ICER values and sum up the results. This review also suggests the need for improvement in several aspects of published cost effectiveness analysis [37].

Only five of the thirteen studies included in the review mentioned target population. Overall, majority of the studies (8/13) mention the sample size but adequate description of the characteristics of the base population is not clearly stated. Although the sample size varied considerably, the authors did not provide the value of standard deviation of average costs. However, these studies represent developed and developing nations as well as low and high TB burden countries. The conclusions of all included studies suggest the generalizability of the observation. Similarly, a systematic review on methodological issues on cost-effectiveness study has also mentioned inadequate reporting of characteristics of the target population which is important for generalizability of the results for decision making [38].

While the cost-effectiveness of implementing a new tool (LED-FM or GeneXpert) is one dimension; the other dimension of clinical effectiveness is considering the sensitivity and specificity for each of the methods. A systematic review conducted on clinical effectiveness of GeneXpert showed that GeneXpert has higher sensitivity than the ZN microscopy. Test accuracy was retained; a single GeneXpert MTB/RIF test directly on sputum detected 99% of smear-positive patients and 80% of patients with smear-negative disease. Thus, GeneXpert is cost effective with increase in sensitivity [39]. It also provides additional information on drug susceptibility of rifampicin.

Of the included studies for GeneXpert, majority were done in South Africa (5/9) [31, 32, 34]. Since South Africa has adopted GeneXpert as an upfront diagnostic for TB, which made it possible for more studies to be conducted. One multi-centric study done in 2012 [34] including South Africa reported cost per sample was USD 45. In the same year (2012) another study was conducted only in South Africa reported cost per sample was USD 21.6 [32]. Though this study did not report the country wise costs, the higher cost may be due to the pooled estimate (due to multi-centric nature of the study). Another study conducted in South Africa in 2016 reported the cost per sample was USD 14.45; indicating that, over a period of time, implementation of GeneXpert seems to be getting more cost-effective [30].

None of these studies considered the patient benefits through GeneXpert to calculate costs-effectiveness. It was reported that average time to detection was less than one day for GeneXpert, one day for microscopy, 17 days for liquid culture and more than 30 days for solid culture. Further, rifampicin resistance was detected in less than one day with GeneXpert compared with an average of 75 days for phenotypic drug sensitive profile. When GeneXpert results were not used to direct therapy, smear-negative TB patients were initiated with treatment in 58 days on an average, as compared to four days when GeneXpert results were used [40]. This has an impact on quality of life of TB patients and leads to increase in QALYs. Moreover, early diagnosis and initiation of treatment will also contribute in reduction of TB transmission. A study from Brazil reported that 35% reduction in TB-related mortality with less advanced disease among the smear-negative patients diagnosed by GeneXpert [41]. However, this aspect is also not considered for the calculation of cost-effectiveness. If all these parameters are taken into consideration for the cost-effectiveness estimation, GeneXpert will be more cost-effective than currently estimated for the diagnosis of pulmonary TB.

### Limitations of the review

In this review, we did not include unpublished studies or studies published in non-indexed journals. The heterogeneity of the included studies in terms of study design, outcome measures limited the scope for synthesising the data and interpretation.

## Conclusion

Our review identifies a consistent trend of the cost effectiveness of LED-FM and GeneXpert in different countries with diverse context of socio-economic condition, HIV burden and geographical distribution. However, all the studies used different parameters to estimate the impact of these tools and this underscores the need for improving the methodological issues related to the conduct and reporting of cost-effectiveness studies.

## Supporting information

S1 Table PRISMA checklist. (DOCX)

S2 Table Search strategy used to search various databases (PubMed/MEDLINE/EMBASE/Cochran/CEA Registry). (DOCX)

S3 Table List of references excluded due to non-ZN comparator. (DOCX)

S4 Table List of references excluded due to non-reporting of effectiveness indicator. (DOCX)

## References

[1] World Health Organization. Global Tuberculosis Report 2016. Geneva 2016.

[2] World Health Organization. Gear up to End TB: Introducing the End TB Strategy. Geneva 2015.

[3] World Health Organization. Global Tuberculosis Report 2015. Geneva 2015.

[4] World Health Organization. Early detection of Tuberculosis: An overview of approaches, guidelines and tools. Geneva 2011.

[5] World Health Organization, Regional Office for South East Asia. Scale up TB control initiatives to reach the missing one million cases. 2015.

[6] Laokri S, Drabo MK, Weil O, Kafando B, Dembele SM, Dujardin B. Patients Are Paying Too Much for Tuberculosis: A Direct Cost-Burden Evaluation in Burkina Faso. PLoS One. 2013;8(2):e56752 10.1371/journal.pone.0056752 23451079 PMC3581516

[7] Molicotti P, Bua A, Zanetti S. Cost-effectiveness in the diagnosis of tuberculosis: choices in developing countries. J Infect Dev Ctries. 2014;8(1):24–38. 10.3855/jidc.3295 24423709

[8] World Health Organization. Fluorescent light-emitting diode (LED) microscopy for diagnosis of tuberculosis: policy statement. Geneva, 2011. 23586120

[9] World Health Organization. Implementing tuberculosis diagnostics: A policy framework. Geneva 2015.

[10] World Health Organization. Policy Statement: Automated Real-Time Nucleic Acid Amplification Technology for Rapid and Simultaneous Detection of Tuberculosis and Rifampicin Resistance: Xpert MTB/RIF System. Geneva 2011. 26158191

[11] Kanabus A. Information about Tuberculosis. Global Health Education 2017 TBFacts.org

[12] World Health Organization. WHO endorses new rapid tuberculosis test: A major milestone for global TB diagnosis and care. World Health Organization, Geneva, 2010.

[13] Kelly V, Sagili KD, Satyanarayana S, Reza LW, Chadha SS, Wilson NC. Cost-utility analysis of LED fluorescence microscopy in the diagnosis of pulmonary tuberculosis in Indian settings. Int J Tuberc Lung Dis. 2015;19(6):696–701. 10.5588/ijtld.14.0203 25946362

[14] Central TB Division. TB India 2017—Annual Status Report. Directorate General of Health Services, Government of India, Ministry of Health and Family Welfare, New Delhi, India 2017

[15] Kaur R, Kachroo K, Sharma JK, Vatturi SM, Dang A. Diagnostic accuracy of xpert test in tuberculosis detection: A systematic review and meta-analysis. J Global Infect Dis. 2016;8(1):32. 10.4103/0974-777X.176143 PMC4785755 27013842

[16] Reynolds MR, Magnuson EA, Lei Y, Wang K, Vilain K, Li H, et al Cost-effectiveness of transcatheter aortic valve replacement compared with surgical aortic valve replacement in high-risk patients with severe aortic stenosis: results of the PARTNER (Placement of Aortic Transcatheter Valves) trial (Cohort A). J Am Coll Cardiol 2012;60:2683–92. 10.1016/j.jacc.2012.09.018 23122802

[17] Yan L, Xiao H, Zhang Q. Systematic review: Comparison of Xpert MTB/RIF, LAMP and SAT methods for the diagnosis of pulmonary tuberculosis. Tuberculosis. 2016;96:75–86. 10.1016/j.tube.2015.11.005 26786658

[18] Liberati A, Altman DG, Tetzlaff J, Mulrow C, Gøtzsche PC, Ioannidis JP, et al The PRISMA statement for reporting systematic reviews and meta-analyses of studies that evaluate health care interventions: explanation and elaboration. PLoS Med. 2009;6(7):e1000100 10.1371/journal.pmed.1000100 19621070 PMC2707010

[19] Sagili K, Shringarpure K, Nilgiriwala K, Muniyandi M. Cost-effectiveness of GeneXpert, LED FM and chest X-ray for diagnosis of pulmonary tuberculosis: a systematic review and meta-analysis. PROSPERO 2016:CRD42016043333 Available from http://www.crd.york.ac.uk/PROSPERO_REBRANDING/display_record.asp?ID=CRD42016043333 10.1371/journal.pone.0205233 PMC6205591 30372436

[20] Pang F, Drummond M, Song F. The use of meta-analysis in economic evaluation. Centre for Health Economics, University of York, 1999.

[21] Tufts Medical Center. Cost effectiveness analysis registry. http://healtheconomics.tuftsmedicalcenter.org/cear4/

[22] Cochrane Library. Cochrane Database of Systematic Reviews. http://onlinelibrary.wiley.com/cochranelibrary/search

[23] Ouzzani M, Hammady H, Fedorowicz Z, Elmagarmid A. Rayyan—a web and mobile app for systematic reviews. Systematic reviews. 2016;5(1):210 10.1186/s13643-016-0384-4 27919275 PMC5139140

[24] Odnoletkova I, Goderis G, Pil L, Nobels F, Aertgeerts B, Annemans L, et al Cost-Effectiveness of Therapeutic Education to Prevent the Development and Progression of Type 2 Diabetes. Systematic Review. J Diab Metab. 2014;5(9).

[25] Whitelaw A, Peter J, Sohn H, Viljoen D, Theron G, Badri M, et al Comparative cost and performance of light-emitting diode microscopy in HIV-tuberculosis-co-infected patients. Eur Res J. 2011;38(6):1393–7. 10.1183/09031936.00023211 PMC5454486 21659413

[26] Sohn H, Sinthuwattanawibool C, Rienthong S, Varma JK. Fluorescence microscopy is less expensive than Ziehl-Neelsen microscopy in Thailand. Int J Tuberc Lung Dis 2009;13(2):266–8. 19146758

[27] Xia H, Song YY, Zhao B, Kam KM, O'Brien RJ, Zhang ZY, et al Multicentre evaluation of Ziehl-Neelsen and light-emitting diode fluorescence microscopy in China. Int J Tuberc Lung Dis 2013;17(1):107–112. 10.5588/ijtld.12.0184 23232010

[28] Mishra L, Henostroza G, Harris J, Siyambango M, Krunner A, Kaunda K, et al Evaluating the cost-effectiveness of TB diagnostic strategies in HIV-positive patients in Lusaka, Zambia. Int J of Infect Dis 2012;16: e286.

[29] Pinto M, Steffen RE, Cobelens F, van den Hof S, Entringer A, Trajman A. Cost-effectiveness of the Xpert(R) MTB/RIF assay for tuberculosis diagnosis in Brazil. Int J Tuberc Lung Dis. 2016;20(5):611–8. 10.5588/ijtld.15.0455 27084814

[30] You JH, Lui G, Kam KM, Lee NL. Cost-effectiveness analysis of the Xpert MTB/RIF assay for rapid diagnosis of suspected tuberculosis in an intermediate burden area. J Infect. 2015;70(4):409–14. 10.1016/j.jinf.2014.12.015 25573001

[31] Jha S, Ismail N, Clark D, Lewis JJ, Omar S, Dreyer A, et al Cost-Effectiveness of Automated Digital Microscopy for Diagnosis of Active Tuberculosis. PloS One. 2016;11(6):e0157554 10.1371/journal.pone.0157554 27322162 PMC4913947

[32] Andrews JR, Lawn SD, Rusu C, Wood R, Noubary F, Bender MA, et al The cost-effectiveness of routine tuberculosis screening with Xpert MTB/RIF prior to initiation of antiretroviral therapy: a model-based analysis. AIDS. 2012;26(8):987–95. 10.1097/QAD.0b013e3283522d47 22333751 PMC3517815

[33] Millman AJ, Dowdy DW, Miller CR, Brownell R, Metcalfe JZ, Cattamanchi A, et al Rapid molecular testing for TB to guide respiratory isolation in the U.S.: a cost-benefit analysis. PloS One. 2013;8(11):e79669 10.1371/journal.pone.0079669 24278155 PMC3835836

[34] Menzies NA, Cohen T, Lin HH, Murray M, Salomon JA. Population health impact and cost-effectiveness of tuberculosis diagnosis with Xpert MTB/RIF: a dynamic simulation and economic evaluation. PLoS Med. 2012;9(11):e1001347 10.1371/journal.pmed.1001347 23185139 PMC3502465

[35] Tesfaye A, Fiseha D, Assefa D, Klinkenberg E, Balanco S, Langley I. Modeling the patient and health system impacts of alternative xpert(R) MTB/RIF algorithms for the diagnosis of pulmonary tuberculosis in Addis Ababa, Ethiopia. BMC Infect Dis. 2017;17(1):318 10.1186/s12879-017-2417-6 28464797 PMC5414345

[36] Vassall A, Siapka M, Foster N, Cunnama L, Ramma L, Fielding K, et al Cost-effectiveness of Xpert MTB/RIF for tuberculosis diagnosis in South Africa: a real-world cost analysis and economic evaluation. Lan Glob Health. 2017;5(7):e710–e9. 10.1016/S2214-109X(17)30205-X PMC5471605 28619229

[37] Wong CK, Liao Q, Guo VY, Xin Y, Lam CLK. Cost-effectiveness analysis of vaccinations and decision makings on vaccination programmes in Hong Kong: A systematic review. Vaccine 2017, 35(24):3153–3161. 10.1016/j.vaccine.2017.04.050 28476628

[38] Catalá-López F, Ridao M, Alonso-Arroyo A, García-Altés A, Cameron C, González-Bermejo D, et al The quality of reporting methods and results of cost-effectiveness analyses in Spain: a methodological systematic review. Systematic reviews. 2016;5(1):6. 26822374 10.1186/s13643-015-0181-5 PMC4731991

[39] Dinnes J, Deeks J, Kunst H, Gibson A, Cummins E, Waugh N, et al A systematic review of rapid diagnostic tests for the detection of tuberculosis infection. Health Technology Assessment Southampton 2007;11(3). 10.3310/hta11030 17266837

[40] World Health Organization. Xpert MTB/RIF implementation manual: Technical and operational ‘how-to’: practical considerations. Geneva, Switzerland: WHO, 2014. 25473699

[41] Trajman A, Durovni B, Saraceni V, Menezes A, Cordeiro-Santos M, Cobelens F, et al Impact on patients’ treatment outcomes of XpertMTB/RIF implementation for the diagnosis of tuberculosis: follow-up of a stepped-wedge randomized clinical trial. PloS One. 2015;10(4):e0123252 10.1371/journal.pone.0123252 25915745 PMC4411054

