# Supplementary material for: Cost-effectiveness of GeneXpert and LED-FM for diagnosis of pulmonary tuberculosis: A systematic review
Source: PLoS One. 2018 Oct 29;13(10):e0205233. doi: 10.1371/journal.pone.0205233 (PMC6205591; doi:10.1371/journal.pone.0205233)
Supplement: S2 Table — (DOCX) [file pone.0205233.s002.docx]

**Table S2. Search strategy used to search various databases PubMed/MEDLINE/EMBASE/Cochran/CEA Registry)**

| **Disease terms:** (Tuberculosis[MeSH] or tuberculosis or TB or tb or Koch’s) AND (Diagnosis or screening or tests or diagnostics) AND (sputum microscopy or ZN microscopy or Ziehl Neelsen or AFB staining or Acid fast bacilli staining or mtb staining or sputum smear or LED fluorescence microscopy or FM staining or LED-FM or Auramine staining or GeneXpert or Xpert or MTB/RIF or CBNAAT or Xpert MTB/RIF assay or Cepheid OR Chest X-ray or MMR or mass miniature radiography or Mass chest X-ray or Roentgenography or radiography or diagnostic X-ray or digital X-ray) AND (Cost effectiveness or cost benefit analysis or cost utility analysis or costs and benefits or economic evaluation) NOT (extra-pulmonary) NOT (EPTB) |
| --- |
| **Tool terms & Cost Effectiveness terms:**  Strategy-1: TB + diagnostic tools + cost effectiveness; tuberculosis AND diagnostic tools AND cost effectiveness  Strategy-2: TB + microscopy + cost effectiveness; tuberculosis AND microscopy AND cost effectiveness.  Strategy-3: TB + fluorescence microscopy + cost effectiveness; tuberculosis AND fluorescence microscopy AND cost effectiveness  Strategy-4: TB + GeneXpert + cost effectiveness; tuberculosis AND GeneXpert AND cost effectiveness  Strategy-5: TB + X-ray + cost effectiveness; tuberculosis AND X-ray AND cost effectiveness  Strategy-6: TB + sputum microscopy + cost effectiveness; tuberculosis + sputum microscopy + cost effectiveness  Strategy-7: TB + LED-FM + cost effectiveness; tuberculosis + LED-FM + cost effectiveness  Strategy-8: TB + GeneXpert + cost effectiveness; tuberculosis + GeneXpert + cost effectiveness  Strategy-9: TB + Chest X-ray + cost effectiveness; tuberculosis + Chest X-ray + cost effectiveness  Strategy-10: TB + sputum microscopy + LED-FM + GeneXpert + Chest X-ray + cost effectiveness; tuberculosis + sputum microscopy + LED-FM + GeneXpert + Chest X-ray + cost effectiveness |
