# Supplementary material for: Cost-effectiveness of GeneXpert and LED-FM for diagnosis of pulmonary tuberculosis: A systematic review
Source: PLoS One. 2018 Oct 29;13(10):e0205233. doi: 10.1371/journal.pone.0205233 (PMC6205591; doi:10.1371/journal.pone.0205233)
Supplement: S3 Table — (DOCX) [file pone.0205233.s003.docx]

**Table S3: List of references excluded due to non-ZN comparator**

| 1. Albert H (2004) Economic analysis of the diagnosis of smear-negative pulmonary tuberculosis in South Africa: incorporation of a new rapid test, FASTPlaqueTB, into the diagnostic algorithm. The international journal of tuberculosis and lung disease : the official journal of the International Union against Tuberculosis and Lung Disease 8: 240-247. |
| --- |
| 1. Azadi M, Bishai DM, Dowdy DW*, et al.* (2014) Cost-effectiveness of tuberculosis screening and isoniazid treatment in the TB/HIV in Rio (THRio) Study. *The international journal of tuberculosis and lung disease : the official journal of the International Union against Tuberculosis and Lung Disease* **18**: 1443-1448. |
| 1. Bonnet M, Tajahmady A, Hepple P, Ramsay A, Githui W, Gagdnidze L, Guerin PJ & Varaine F (2010) Added value of bleach sedimentation microscopy for diagnosis of tuberculosis: a cost-effectiveness study. *The international journal of tuberculosis and lung disease : the official journal of the International Union against Tuberculosis and Lung Disease* **14**: 571-577. |
| 1. Choi HW, Miele K, Dowdy D & Shah M (2013) Cost-effectiveness of Xpert(R) MTB/RIF for diagnosing pulmonary tuberculosis in the United States. *The international journal of tuberculosis and lung disease : the official journal of the International Union against Tuberculosis and Lung Disease* **17**: 1328-1335. |
| 1. Dasgupta K & Menzies D (2005) Cost-effectiveness of tuberculosis control strategies among immigrants and refugees. *The European respiratory journal* **25**: 1107-1116. |
| 1. Dowdy DW, O'Brien MA & Bishai D (2008) Cost-effectiveness of novel diagnostic tools for the diagnosis of tuberculosis. *The international journal of tuberculosis and lung disease : the official journal of the International Union against Tuberculosis and Lung Disease* **12**: 1021-1029. |
| 1. Dowdy DW, Steingart KR & Pai M (2011) Serological testing versus other strategies for diagnosis of active tuberculosis in India: a cost-effectiveness analysis. *PLoS medicine* **8**: e1001074. |
| 1. Doyle P, Wong T, Roscoe D, Connolly E & Koeck E (2015) Xpert MTB/RIF as a supplemental test to acid-fast bacili sputum microscopy in the diagnosis of tuberculosis: Potential resource savings. *Canadian Journal of Infectious Diseases and Medical Microbiology* **26**: e47. |
| 1. Eang MT, Satha P, Yadav RP, Morishita F, Nishikiori N, van-Maaren P & Weezenbeek CL (2012) Early detection of tuberculosis through community-based active case finding in Cambodia. *BMC public health* **12**: 469. |
| 1. Hausler HP, Sinanovic E, Kumaranayake L, Naidoo P, Schoeman H, Karpakis B & Godfrey-Faussett P (2006) Costs of measures to control tuberculosis/HIV in public primary care facilities in Cape Town, South Africa. *Bulletin of the World Health Organization* **84**: 528-536. |
| 1. Hsiang E, Little KM, Haguma P, Hanrahan CF, Katamba A, Cattamanchi A, Davis JL, Vassall A & Dowdy D (2016) Higher cost of implementing Xpert((R)) MTB/RIF in Ugandan peripheral settings: implications for cost-effectiveness. *The international journal of tuberculosis and lung disease : the official journal of the International Union against Tuberculosis and Lung Disease* **20**: 1212-1218. |
| 1. Hughes R, Wonderling D, Li B & Higgins B (2012) The cost effectiveness of Nucleic Acid Amplification Techniques for the diagnosis of tuberculosis. *Respiratory medicine* **106**: 300-307. |
| 1. Jones TF & Schaffner W (2001) Miniature chest radiograph screening for tuberculosis in jails: a cost-effectiveness analysis. *Am J Respir Crit Care Med* **164**: 77-81. |
| 1. Kowada A (2010) Cost effectiveness of IGRAs assays in Japan. *International Journal of Tuberculosis and Lung Disease* **14**: S59-S60. |
| 1. Kranzer K, Lawn SD, Meyer-Rath G, Vassall A, Raditlhalo E, Govindasamy D, van Schaik N, Wood R & Bekker LG (2012) Feasibility, yield, and cost of active tuberculosis case finding linked to a mobile HIV service in Cape Town, South Africa: a cross-sectional study. *PLoS medicine* **9**: e1001281. |
| 1. Langley I, Lin HH, Egwaga S, Doulla B, Ku CC, Murray M, Cohen T & Squire SB (2014) Assessment of the patient, health system, and population effects of Xpert MTB/RIF and alternative diagnostics for tuberculosis in Tanzania: an integrated modelling approach. *The Lancet Global health* **2**: e581-591. |
| 1. Lee KK, Fun WH, Wu DB, Cheong YM & Mohamad Noordin N (2016) Cost-effectiveness analysis of Xpert MTB/RIF assay for diagnosing tuberculosis in Malaysia using dynamic transmission model. *Value in Health* **19**: A859. |
| 1. Maheswaran H & Barton P (2012) Intensive case finding and isoniazid preventative therapy in HIV infected individuals in Africa: economic model and value of information analysis. *PLoS One* **7**: e30457. |
| 1. Munoz L, Moure R, Porta N, Gonzalez L, Guerra R, Alcaide F & Santin M (2013) GeneXpert(R) for smear-negative pulmonary tuberculosis: does it play a role in low-burden countries? *Diagnostic microbiology and infectious disease* **75**: 325-326. |
| 1. Perlman DC, Gourevitch MN, Trinh C, Salomon N, Horn L & Des Jarlais DC (2001) Cost-effectiveness of tuberculosis screening and observed preventive therapy for active drug injectors at a syringe-exchange program. *Journal of urban health : bulletin of the New York Academy of Medicine* **78**: 550-567. |
| 1. Pho MT, Deo S, Palamountain KM, Joloba ML, Bajunirwe F & Katamba A (2015) Optimizing tuberculosis case detection through a novel diagnostic device placement model: the case of Uganda. *PLoS One* **10**: e0122574. |
| 1. Rajalahti I, Ruokonen EL, Kotomaki T, Sintonen H & Nieminen MM (2004) Economic evaluation of the use of PCR assay in diagnosing pulmonary TB in a low-incidence area. *The European respiratory journal* **23**: 446-451. |
| 1. Samandari T, Bishai D, Luteijn M, Mosimaneotsile B, Motsamai O, Postma M & Hubben G (2011) Costs and consequences of additional chest x-ray in a tuberculosis prevention program in Botswana. *Am J Respir Crit Care Med* **183**: 1103-1111. |
| 1. Scherer LC, Sperhacke RD, Ruffino-Netto A, Rossetti ML, Vater C, Klatser P & Kritski AL (2009) Cost-effectiveness analysis of PCR for the rapid diagnosis of pulmonary tuberculosis. *BMC Infect Dis* **9**: 216. |
| 1. Schwartzman K & Menzies D (2000) Tuberculosis screening of immigrants to low-prevalence countries. A cost-effectiveness analysis. *Am J Respir Crit Care Med* **161**: 780-789. |
| 1. Shah M, Dowdy D, Joloba M, Ssengooba W, Manabe YC, Ellner J & Dorman SE (2013) Cost-effectiveness of novel algorithms for rapid diagnosis of tuberculosis in HIV-infected individuals in Uganda. *AIDS (London, England)* **27**: 2883-2892. |
| 1. Suen SC, Bendavid E & Goldhaber-Fiebert JD (2015) Cost-effectiveness of improvements in diagnosis and treatment accessibility for tuberculosis control in India. *The international journal of tuberculosis and lung disease : the official journal of the International Union against Tuberculosis and Lung Disease* **19**: 1115-1124, i-xv. |
| 1. van't Hoog AH, Cobelens F, Vassall A, van Kampen S, Dorman SE, Alland D & Ellner J (2013) Optimal triage test characteristics to improve the cost-effectiveness of the Xpert MTB/RIF assay for TB diagnosis: a decision analysis. *PLoS One* **8**: e82786. |
| 1. Verma G, Chuck AW & Jacobs P (2013) Tuberculosis screening for long-term care: a cost-effectiveness analysis. *The international journal of tuberculosis and lung disease : the official journal of the International Union against Tuberculosis and Lung Disease* **17**: 1170-1177. |
| 1. Walker D, McNerney R, Mwembo MK, Foster S, Tihon V & Godfrey-Faussett P (2000) An incremental cost-effectiveness analysis of the first, second and third sputum examination in the diagnosis of pulmonary tuberculosis. *The international journal of tuberculosis and lung disease : the official journal of the International Union against Tuberculosis and Lung Disease* **4**: 246-251. |
| 1. Yakhelef N, Audibert M, Varaine F, Chakaya J, Sitienei J, Huerga H & Bonnet M (2014) Is introducing rapid culture into the diagnostic algorithm of smear-negative tuberculosis cost-effective? *The international journal of tuberculosis and lung disease : the official journal of the International Union against Tuberculosis and Lung Disease* **18**: 541-546. |
| 1. Zishiri V, Charalambous S, Shah MR, Chihota V, Page-Shipp L, Churchyard GJ & Hoffmann CJ (2015) Implementing a large-scale systematic tuberculosis screening program in correctional facilities in South Africa. *Open forum infectious diseases* **2**: ofu121. |
| 1. Zwerling A, White RG, Vassall A, Cohen T, Dowdy DW & Houben RM (2014) Modeling of novel diagnostic strategies for active tuberculosis - a systematic review: current practices and recommendations. *PLoS One* **9**: e110558. |
| 1. Zwerling AA, Sahu M, Ngwira LG, Khundi M, Harawa T, Corbett EL, Chaisson RE & Dowdy DW (2015) Screening for Tuberculosis Among Adults Newly Diagnosed With HIV in Sub-Saharan Africa: A Cost-Effectiveness Analysis. *Journal of acquired immune deficiency syndromes (1999)* **70**: 83-90. |
