# Supplementary material for: Cost-effectiveness of GeneXpert and LED-FM for diagnosis of pulmonary tuberculosis: A systematic review
Source: PLoS One. 2018 Oct 29;13(10):e0205233. doi: 10.1371/journal.pone.0205233 (PMC6205591; doi:10.1371/journal.pone.0205233)
Supplement: S4 Table — (DOCX) [file pone.0205233.s004.docx]

**Table S4: List of references excluded due to non-reporting of effectiveness indicator**

| 1. Abimbola TO, Marston BJ, Date AA, Blandford JM, Sangrujee N & Wiktor SZ (2012) Cost-effectiveness of tuberculosis diagnostic strategies to reduce early mortality among persons with advanced HIV infection initiating antiretroviral therapy. *Journal of acquired immune deficiency syndromes (1999)* **60**: e1-7. |
| --- |
| 1. Aliyu G, El-Kamary SS, Abimiku A, Hungerford L, Obasanya J & Blattner W (2014) Cost-effectiveness of point-of-care digital chest-x-ray in HIV patients with pulmonary mycobacterial infections in Nigeria. *BMC Infect Dis* **14**: 675. |
| 1. Cunnama L, Sinanovic E, Ramma L, Foster N, Berrie L, Stevens W, Molapo S, Marokane P, McCarthy K & Churchyard G (2016) Using top‐down and bottom‐up costing approaches in LMICs: The case for using both to assess the incremental costs of new technologies at scale. *Health economics* **25**: 53-66. |
| 1. Diel R, Nienhaus A, Hillemann D & Richter E (2015) Cost–benefit analysis of Xpert® MTB/RIF for tuberculosis suspects in German hospitals. *European Respiratory Journal* ERJ-01333-02015. |
| 1. Fitzgerald D, Jansen P, Chipungu C, Dindi V, Fielder J & Pfaff C (2012) High cost of Xpert MTB/RIF testing per excess tuberculosis case diagnosed at partners in hope medical Center, a public-private HIV care clinic in Lilongwe, Malawi. Comparison with fluorescence microscopy in a wellequipped and experienced real world AFB laboratory. *Journal of the International AIDS Society* **15**: 44. |
| 1. Guerra RL, Dorman SE, Luiz RR & Conde MB (2013) Cost-effectiveness of routine diagnostic evaluation of pulmonary tuberculosis in a primary care unit in Brazil. *The international journal of tuberculosis and lung disease : the official journal of the International Union against Tuberculosis and Lung Disease* **17**: 1336-1340. |
| 1. Gupta S, Abimbola T, Date A, Suthar AB, Bennett R, Sangrujee N & Granich R (2014) Cost-effectiveness of the Three I's for HIV/TB and ART to prevent TB among people living with HIV. *The international journal of tuberculosis and lung disease : the official journal of the International Union against Tuberculosis and Lung Disease* **18**: 1159-1165. |
| 1. Ho J, Jelfs P, Nguyen P, Sintchenko V, Fox G & Marks G (2017) Pooling sputum samples to improve the feasibility of Xpert® MTB/RIF in systematic screening for tuberculosis. *The International Journal of Tuberculosis and Lung Disease* **21**: 503-508. |
| 1. Kivihya-Ndugga LE, van Cleeff MR, Githui WA, Nganga LW, Kibuga DK, Odhiambo JA & Klatser PR (2003) A comprehensive comparison of Ziehl-Neelsen and fluorescence microscopy for the diagnosis of tuberculosis in a resource-poor urban setting. *The international journal of tuberculosis and lung disease : the official journal of the International Union against Tuberculosis and Lung Disease* **7**: 1163-1171. |
| 1. Naidoo P, Dunbar R, Du Toit E, Van Niekerk M, Squire SB, Beyers N & Madan J (2016) Comparing laboratory costs of smear/culture and Xpert® MTB/RIF-based tuberculosis diagnostic algorithms. *International Journal of Tuberculosis and Lung Disease* **20**: 1377-1385. |
| 1. Pantoja A, Fitzpatrick C, Vassall A, Weyer K & Floyd K (2013) Xpert MTB/RIF for diagnosis of tuberculosis and drug-resistant tuberculosis: A cost and affordability analysis. *European Respiratory Journal* **42**: 708-720. |
| 1. Pinto M, Entringer AP, Steffen R & Trajman A (2015) Cost analysis of nucleic acid amplification for diagnosing pulmonary tuberculosis, within the context of the Brazilian Unified Health Care System. *Jornal brasileiro de pneumologia : publicacao oficial da Sociedade Brasileira de Pneumologia e Tisilogia* **41**: 536-538. |
| 1. Rupert S, Vassall A, Raizada N, Khaparde S, Boehme C, Salhotra V, Sachdeva K, Nair S & van't Hoog A (2017) Bottom-up or top-down: unit cost estimation of tuberculosis diagnostic tests in India. *The international journal of tuberculosis and lung disease* **21**: 375-380 |
| 1. Schnippel K, Meyer-Rath G, Long L, MacLeod W, Sanne I, Stevens WS & Rosen S (2012) Scaling up Xpert MTB/RIF technology: the costs of laboratory- vs. clinic-based roll-out in South Africa. *Tropical medicine & international health : TM & IH* **17**: 1142-1151. |
| 1. van Cleeff M, Kivihya-Ndugga L, Githui W, Ng'ang'a L, Kibuga D, Odhiambo J & Klatser P (2005) Cost-effectiveness of polymerase chain reaction versus Ziehl-Neelsen smear microscopy for diagnosis of tuberculosis in Kenya. *The international journal of tuberculosis and lung disease : the official journal of the International Union against Tuberculosis and Lung Disease* **9**: 877-883. |
| 1. Van Rie A, Page-Shipp L, Hanrahan CF, Schnippel K, Dansey H, Bassett J, Clouse K, Scott L, Stevens W & Sanne I (2013) Point-of-care Xpert(R) MTB/RIF for smear-negative tuberculosis suspects at a primary care clinic in South Africa. *The international journal of tuberculosis and lung disease : the official journal of the International Union against Tuberculosis and Lung Disease* **17**: 368-372. |
| 1. Vassall A, van Kampen S, Sohn H*, et al.* (2011) Rapid diagnosis of tuberculosis with the Xpert MTB/RIF assay in high burden countries: a cost-effectiveness analysis. *PLoS medicine* **8**: e1001120. |
| 1. Wang WB, Wang FD, Xu B, Zhu JF, Shen W, Xiao XR & Jiang QW (2006) [A cost-effectiveness study on a case-finding program of tuberculosis through screening those suspects with chronic cough symptoms in the rich rural areas]. *Zhonghua liu xing bing xue za zhi = Zhonghua liuxingbingxue zazhi* **27**: 857-860. |
| 1. Winetsky DE, Negoescu DM, DeMarchis EH, Almukhamedova O, Dooronbekova A, Pulatov D, Vezhnina N, Owens DK & Goldhaber-Fiebert JD (2012) Screening and rapid molecular diagnosis of tuberculosis in prisons in Russia and Eastern Europe: a cost-effectiveness analysis. *PLoS medicine* **9**: e1001348. |
| 1. You J, Lui G, Kam KM & Lee N (2014) Xpert Mtb/Rif Assay for Rapid Diagnosis in Patients with Suspected Tuberculosis in Hong Kong - a Cost-Effectiveness Analysis. *Value in health : the journal of the International Society for Pharmacoeconomics and Outcomes Research* **17**: A678. |
